# Outbreak of Seoul Virus Among Rats and Rat Owners — United States and Canada, 2017

**DOI:** 10.15585/mmwr.mm6704a5

**Published:** 2018-02-02

**Authors:** Janna L. Kerins, Sarah E. Koske, James Kazmierczak, Connie Austin, Karen Gowdy, Antonia Dibernardo, Seoul Virus Working Group, Canadian Seoul Virus Investigation Group (Federal), Canadian Seoul Virus Investigation Group (Provincial), Contributors, Jan Achenbach, Jill Baber, Gary Balsamo, Casey Barton Behravesh, David Blythe, Iwona Boraks-Pielechaty, Shelley M. Brown, Jennifer Brown, Robert Brucker, Barbara A. Bruen, Colin Campbell, Deborah Cannon, Beth Carlson, Kris Carter, Cynthia Cary, Caroline Castillo, Cheng-Feng Chiang, Mary Choi, Ellen Christel, April Clayton, Leah Colton, Laura Cronquist, David Damsker, William G. Davis, Annabelle de St Maurice, Jordan Dieckman, John Dunn, Drew D. Dycus, Elizabeth Ervin, Michelle Feist, Amanda Feldpausch, Mary Margaret Fill, Julie Gabel, Ann Garvey, Sarah Genzer, Suzanne Gibbons-Burgener, James Graziano, Victoria Hall, Russel Heisey, Heather Henderson, Janemarie Hennebelle, Leslie Hiber, Stacy Holzbauer, Jennifer House, Eddie Jackson, Mary H. Jenks, Dee Jones CDC, Susan Keller, John D. Klena, Rachel F. Klos, Barbara Knust, Anna Kocharian, Katrin Kohl, Gregory Langham, George Lathrop, Jennifer Layden, Kathryn Lehatto, Jodi Lovejoy, Kenneth Lowery, Nicole Lukovsky-Akhsanov, Nhiem Luong, Michael Maglio, Craig Manning, Chandra Marriott, Natalie Marzec, Michel Masters, Susan McClanahan, Lisa McCloskey, Shannon McKnight, Jennifer McQuiston, Sara McReynolds, Gianna Megaro, Maria Morales-Betoulle, Allyn K. Nakashima, Stuart Nichol, Julie Paoline, Nishi Patel, Ketan Patel, Dallin Peterson, Leah Posivak-Khouly, Nathaniel Powell, Howard Pue, Lawrence Purpura, Rachel Radcliffe, Nicole Reynolds, Linda Roesch, Pierre Rollin, Andrea L. Sandberg, Joni Scheftel, Betsy Schroeder, Irshad A. Shaikh, Trevor Shoemaker, Jennifer Sidge, Tom Sidwa, Kim Signs, Amber Singh, Aaron Smee, Danielle Stanek, Mary Grace Stobierski, Anne Straily, Ute Ströher, Cassandra Tansey, Leslie Tengelsen, Beth Thompson, Susan L. Ward, Kimberly Warren, Susan Weinstein, Deborah Weiss, Andre Weltman, Tigist Yirko, Joyce Zuzack, Peter A. Buck, Allen Grolla, Chris Huynh, L. Robbin Lindsay, Courtney Loomer, David Safronetz, Angela Sloan, Jim E. Strong, Joanne R. Tataryn, Linda Vrbova

**Affiliations:** ^1^Epidemic Intelligence Service, CDC; ^2^Chicago Department of Public Health, Illinois; ^3^Wisconsin Department of Health Services; ^4^Illinois Department of Public Health; ^5^Population and Public Health Division, Ontario Ministry of Health and Long-Term Care; ^6^Public Health Agency of Canada.; Chester County Department of Health; North Dakota Department of Health; Louisiana Department of Health; CDC; Maryland Department of Health and Mental Hygiene; Pennsylvania Department of Health; CDC; Indiana State Department of Health; Montgomery County Health Department; Pennsylvania Department of Health; New Jersey Department of Health; CDC; North Dakota Department of Agriculture; CDC; Idaho Department of Health and Welfare; CDC; CDC; Michigan Department of Health and Human Services; CDC; CDC; Manitowoc County Health Department; CDC; Colorado Department of Public Health and Environment; North Dakota Department of Health; Bucks County Health Department; CDC; CDC; Wisconsin Department of Health Services; Tennessee Department of Health; Bucks County Health Department; CDC; North Dakota Department of Health; Georgia Department of Public Health; CDC; Tennessee Department of Health; Georgia Department of Public Health; Iowa Department of Public Health; CDC; Wisconsin Department of Health Services; CDC; CDC; Minnesota Department of Health; Pennsylvania Department of Health; Tennessee Department of Health; Georgia Department of Agriculture; Minnesota Department of Health; CDC; Minnesota Department of Health; Colorado Department of Public Health and Environment; CDC; Alabama Department of Public Health; North Dakota Department of Agriculture; CDC; Wisconsin Department of Health Services; CDC; Wisconsin Department of Health Services; CDC; CDC; CDC; Illinois Department of Public Health; Pennsylvania Department of Health; Indiana State Board of Animal Health; Georgia Department of Public Health; CDC; Delaware Health and Social Services; Chester County Department of Health; CDC; Pennsylvania Department of Health; Colorado Department of Public Health and Environment; Montgomery County Health Department; Minnesota Board of Animal Health; Bucks County Health Department; Pennsylvania Department of Health; CDC; North Dakota Department of Agriculture; Chester County Department of Health; CDC; Utah Department of Health; CDC; Montgomery County Health Department; CDC; CDC; Utah Department of Health; Montgomery County Health Department; CDC; Missouri Department of Health & Senior Services; CDC; , South Carolina Department of Health & Environmental Control; Minneapolis Medical Research Foundation; CDC; CDC; Brown County Health and Human Services; Minnesota Department of Health; CDC; Indiana State Department of Health; Montgomery County Department; CDC; Michigan Department of Health and Human Services; Texas Department of State Health Services; Michigan Department of Health and Human Services; Ohio Department of Health; Pennsylvania Department of Health; Florida Department of Health; Michigan Department of Health and Human Services; CDC; CDC; CDC; Idaho Department of Health and Welfare; Minnesota Board of Animal Health; Bucks County Department of Health; Pennsylvania Department of Health; Arkansas Department of Health; CDC; Wisconsin Department of Health Services; Pennsylvania Department of Health; Pennsylvania Department of Health; Bucks County Department of Health; Public Health Agency of Canada (PHAC; PHAC; PHAC; PHAC; PHAC; PHAC; PHAC; PHAC; PHAC; PHAC

In December 2016, the Wisconsin Department of Health Services (WDHS) notified CDC of a patient hospitalized with fever, leukopenia, elevated transaminases, and proteinuria. The patient owned and operated an in-home rattery, or rat-breeding facility, with approximately 100 Norway rats, primarily bred as pets. A family member developed similar symptoms 4 weeks later, but was not hospitalized. Because both patients were known to have rodent contact, they were tested for hantavirus infections. In January 2017, CDC confirmed recent, acute Seoul virus infection in both patients. An investigation was conducted to identify additional human and rat infections and prevent further transmission. Ultimately, the investigation identified 31 facilities in 11 states with human and/or rat Seoul virus infections; six facilities also reported exchanging rats with Canadian ratteries. Testing of serum samples from 183 persons in the United States and Canada identified 24 (13.1%) with Seoul virus antibodies; three (12.5%) were hospitalized and no deaths occurred. This investigation, including cases described in a previously published report from Tennessee (*1*), identified the first known transmission of Seoul virus from pet rats to humans in the United States and Canada. Pet rat owners should practice safe rodent handling to prevent Seoul virus infection (*2*).

Seoul virus is an Old World hantavirus in the *Bunyaviridae* family. Its natural reservoir is the Norway rat (*Rattus norvegicus*). Rats infected with Seoul virus are asymptomatic, but can transmit the virus to humans through infectious saliva, urine, droppings, or aerosolization from contaminated bedding. Human signs and symptoms range from mild influenza-like illness to hemorrhagic fever with renal syndrome (HFRS). HFRS causes acute renal failure and can result in death; however, asymptomatic Seoul virus infections also occur. Wild Norway rats in the United States have been known to harbor Seoul virus infection (*3*), but transmission to humans is rare (*4*). Seoul virus is not known to spread from person to person. In the United Kingdom, Seoul virus transmission has occurred from pet rats to humans (*5*), but before this outbreak, infections had not been reported in pet rats in the United States or Canada.

## Investigation and Results

After confirming Seoul virus infection in the Wisconsin patients, CDC and WDHS initiated investigations into rat shipments to (trace-back) and from (trace-forward) the rattery to identify suspected and confirmed facilities. Trace-back investigations initially extended back 2 months prior to onset of clinical disease, based on the known maximum incubation period for Seoul virus in humans. As additional confirmed facilities were identified, tracing focused instead on interactions with known infected facilities, sometimes as much as 1 year prior. Suspected facilities included ratteries, homes, or pet stores that sold rats to a confirmed facility (a facility where at least one human or rat tested positive for Seoul virus infection) or housed rats that lived at or comingled with rats from a confirmed facility. Once a suspected facility was identified, local or state health officials interviewed persons with a history of rodent contact associated with the facility about their rat exposure and health history. Additionally, the primary rodent caretaker was interviewed using a standardized questionnaire to identify movement of rats into and out of the facility, including dates and locations where the rats were obtained. Local or state health officials offered laboratory testing for Seoul virus infection to all persons with rodent contact. Officials recommended testing for persons with a history of febrile illness and exposure to rats from a confirmed facility and for rats at suspected and confirmed facilities. Trace-forward and trace-back investigations of rat shipments at confirmed facilities identified additional suspected facilities, which were similarly assessed.

A suspected human case of Seoul virus infection was defined as a febrile illness (recorded temperature >101°F [38.3°C] or subjective history of fever) or an illness clinically compatible with Seoul virus infection (myalgia, headache, renal failure, conjunctival redness, thrombocytopenia, or proteinuria) without laboratory confirmation in a person reporting contact with rats from a confirmed or suspected facility. Human Seoul virus infections were laboratory-confirmed by detection of Seoul virus-specific immunoglobulin M (IgM) and/or immunoglobulin G (IgG) (*6*) antibodies by enzyme-linked immunosorbent assay (ELISA). In the United States, Seoul virus infections in rats were confirmed through detection of viral RNA by reverse transcription–polymerase chain reaction (RT-PCR) and/or IgG ELISA at CDC, or by CDC-validated commercial IgG testing. In Canada, public health officials investigated rat breeding facilities that exported rats to and imported rats from affected U.S. facilities. Seoul virus infection was detected in Canadian rats from breeding facilities using the same serologic and molecular-based protocols described for United States facilities.

By March 16, 2017, trace-forward and trace-back investigations identified approximately 100 suspected facilities in 21 states. Among these, 31 facilities in 11 states* had laboratory-confirmed human or rat infections, including a previously reported household in Tennessee with two confirmed human infections (*1*). Six confirmed facilities in six states (Georgia, Illinois, Missouri, South Carolina, Tennessee, and Utah) reported exchanging rats with Canadian ratteries during their trace-forward and trace-back investigations. A total of 163 persons in the United States and 20 in Canada consented to serologic testing; 17 (10.4%) U.S. residents and one (5.0%) Canadian resident had detectable IgM and IgG antibodies, indicating recent infection, and four (2.5%) U.S. residents and two (10.0%) Canadian residents had only IgG antibodies, indicating past or convalescent infection. Among the 17 U.S. patients with recent Seoul virus infection, eight reported recent febrile illness. Three were hospitalized, but did not develop HFRS, and all recovered. Serious illness was not reported in any Canadian patients. All strains detected in Canada and the United States were indistinguishable from one another based on nucleotide sequencing (*7*), indicating that a single strain was responsible for the outbreak. No single facility was identified as the origin of the outbreak.

## Public Health Response

On January 24, CDC issued a Health Alert Notice to notify health departments and health care providers of the Seoul virus investigations.^†^ On February 20, the World Health Organization was notified of the U.S. and Canadian infections and investigations as required by International Health Regulations.^§^ On January 31 and May 9, 2017, CDC and the Pet Industry Joint Advisory Council hosted calls to provide updates on the Seoul virus outbreak and to answer questions for the pet industry and fancy rat community. CDC created a website with Seoul virus facts and frequently asked questions for the public.

Health departments notified suspected and confirmed facilities and placed those facilities under quarantine, allowing no rats to enter or leave. Rat contact was limited to as few persons as possible to reduce transmission. In suspected facilities, CDC recommended rat testing be performed under the supervision of a public health official or licensed veterinarian. The quarantine was lifted when at least 4 weeks had elapsed since the newest animal was introduced, and all rats subsequently tested negative. Rats belonging to owners who refused to test their animals could remain quarantined for life or be euthanized. CDC recommended euthanasia of all rats in confirmed facilities as the most effective method to prevent transmission, although control recommendations differed by state and country according to local policies and response capacities. If euthanasia was not possible, then owners could either quarantine all rats for life or pursue quarantine with testing and culling. The testing and culling strategy entailed testing all rats and euthanizing only infected rats. Testing and euthanasia were repeated at 4 week intervals until all rats tested negative and the quarantine was lifted. In Canada, public health officials opted for education and a voluntary testing and culling approach to control Seoul virus transmission.

## Discussion

This outbreak report, in parallel to the previously described investigation in Tennessee (*1*), describes the first known cases of Seoul virus infection in humans attributable to contact with pet rats in the United States and Canada. Human hantavirus infections are nationally notifiable in the United States and suspected cases should be reported to state or local health departments. Health care providers should consider Seoul virus infection in patients with febrile illness who report rat exposure; CDC recommends testing for any person with compatible illness and rodent contact. Testing is available at CDC^¶^ and through some state and commercial laboratories. In Canada, testing is available for symptomatic persons with rat exposure, rattery owners associated with this investigation, and their rats through public health laboratories; for individually owned pet rats and ratteries not associated with the investigation, testing is available through a commercial laboratory.

Pet rat owners should be aware of the potential for Seoul virus infection. To keep themselves and their pets healthy, all persons with rodent contact should avoid bites or scratches and practice good hand hygiene, especially children and persons with compromised immune systems (*2*). CDC recommends hand washing after caring for rodents and before eating, drinking, or preparing food (*2*). If a pet rat is suspected of having Seoul virus, the person cleaning the rodent environment should wear a respirator, gloves, and cover any scratches or open wounds (*8*). An adult should routinely disinfect rat cages and accessories, including used bedding, with a 10% bleach solution or a commercial disinfectant (*8*). More information about rodent contact and disease prevention is available from CDC (*8**,**9*).

Rattery owners are encouraged to quarantine any newly acquired rats for 4 weeks and to test these rats for Seoul virus antibodies before allowing them to comingle with other rats. Commercial laboratories can perform Seoul virus testing of rodent blood samples, and comparisons of results from shared samples have been concordant with CDC’s ELISA and RT-PCR assays. To prevent transmission to humans, CDC recommends euthanasia of all rats in facilities with human or rat Seoul virus infections. Further guidance on methods to eradicate Seoul virus from infected ratteries should be obtained from local or state health departments.

**Summary**

**What is already known about this topic?**

Seoul virus, a type of hantavirus, is carried by Norway rats. Humans become infected through contact with virus shed in rat urine or droppings, or inhalation of virus particles in dust from contaminated bedding. Infected rats do not develop disease, but humans can experience symptoms ranging from mild influenza-like illness to severe disease with kidney failure and death. Although infections have been previously reported in humans after contact with wild rats, Seoul virus infections had not been reported in pet rats in the United States or Canada.

**What is added by this report?**

This report describes the first known outbreak of Seoul virus infections in humans from contact with pet rats in the United States and Canada. This investigation identified 31 U.S. facilities with human and/or rat Seoul virus infections in 11 states, including six that exchanged rats with Canadian ratteries. Seventeen persons had recent infection with Seoul virus, eight became ill, and three were hospitalized and recovered.

**What are the implications for public health practice?**

Human hantavirus infections are reportable to state or local health departments in the United States. Clinicians should consider Seoul virus infection in patients with a history of rat contact and compatible symptoms. Pet rat owners and breeders should also be aware of Seoul virus and should practice good hand hygiene and safe rodent handling to prevent infection.

## Footnotes

* Colorado, Georgia, Iowa, Illinois, Minnesota, Missouri, Pennsylvania, South Carolina, Tennessee, Utah, and Wisconsin.

†
https://emergency.cdc.gov/han/han00400.asp .

§
http://www.who.int/csr/don/20-february-2017-seoulvirus-usa-and-canada/en/ .

¶
https://www.cdc.gov/hantavirus/health-care-workers/specimen-submission/index.html .
