# *Erratum*: Vol. 67, No. 4

**DOI:** 10.15585/mmwr.mm6707a7

**Published:** 2018-02-23

In the report “Outbreak of Seoul Virus Among Rats and Rat Owners — United States and Canada, 2017,” on page 132, the second sentence of the first paragraph under “Public Health Response,” should have read “On February **10, 2017,** the World Health Organization was notified of the U.S. and Canadian infections and investigations as required by International Health Regulations.^§^”

